# Comparison of Cefotaxime-Resistant *Escherichia coli* and *sul*1 and *int*I1 by qPCR for Monitoring of Antibiotic Resistance of Wastewater, Surface Water, and Recycled Water

**DOI:** 10.3390/antibiotics12081252

**Published:** 2023-07-29

**Authors:** Krista Liguori, Jeanette Calarco, Gabriel Maldonado Rivera, Anna Kurowski, Ishi Keenum, Benjamin C. Davis, Valerie J. Harwood, Amy Pruden

**Affiliations:** 1 Via Department of Civil and Environmental Engineering, Virginia Tech, Blacksburg, VA 24060, USAgabrielm21@vt.edu (G.M.R.); dbenj95@vt.edu (B.C.D.); 2 Department of Integrative Biology, University of South Florida, Tampa, FL 33620, USAvharwood@usf.edu (V.J.H.)

**Keywords:** antimicrobial resistance, standard methods, wastewater monitoring

## Abstract

Awareness of the need for surveillance of antimicrobial resistance (AMR) in water environments is growing, but there is uncertainty regarding appropriate monitoring targets. Adapting culture-based fecal indicator monitoring to include antibiotics in the media provides a potentially low-tech and accessible option, while quantitative polymerase chain reaction (qPCR) targeting key genes of interest provides a broad, quantitative measure across the microbial community. The purpose of this study was to compare findings obtained from the culture of cefotaxime-resistant (cefR) *Escherichia coli* with two qPCR methods for quantification of antibiotic resistance genes across wastewater, recycled water, and surface waters. The culture method was a modification of US EPA Method 1603 for *E. coli*, in which cefotaxime is included in the medium to capture cefR strains, while qPCR methods quantified *sul*1 and *int*I1. A common standard operating procedure for each target was applied to samples collected by six water utilities across the United States and processed by two laboratories. The methods performed consistently, and all three measures reflected the same overarching trends across water types. The qPCR detection of *sul*1 yielded the widest dynamic range of measurement as an AMR indicator (7-log versus 3.5-log for cefR *E. coli*), while *int*I1 was the most frequently detected target (99% versus 96.5% and 50.8% for *sul*1 and cefR *E. coli*, respectively). All methods produced comparable measurements between labs (*p* < 0.05, Kruskal–Wallis). Further study is needed to consider how relevant each measure is to capturing hot spots for the evolution and dissemination of AMR in the environment and as indicators of AMR-associated human health risk.

## 1. Introduction

Antimicrobial resistance (AMR) is no longer a “coming threat” to global health; the threat has arrived. A comprehensive evaluation of globally available data from 2019 estimated that 4.95 million people die of drug-resistant infections per year [1]. Current rates of morbidity and disability-adjusted life years due to AMR are competing with, if not exceeding, those of the global malaria burden [1]. AMR is not confined to hospitals; as Robinson et al. [2] pointed out, it is the “quintessential One-Health issue”, which showcases the need for a holistic, interconnected human–animal–environment approach to research, prevention, and monitoring.

Key environmental dimensions of AMR are increasingly being recognized, as elaborated upon in a recent United Nations Environment Programme (UNEP) report [3]. Among several recommendations, the UNEP report identifies the need to establish environmental AMR monitoring programs to help inform and evaluate policy aimed at abating the spread of AMR. Similar calls have been made by others (e.g., NASEM [4]), and a recent review summarizes opportunities to expand existing AMR monitoring efforts to include water environments [5]. A major stumbling block to achieving a cohesive surveillance effort is a lack of agreement on targets and methods that are representative of AMR concerns in the environment.

There have been a number of calls for coordinated AMR monitoring in the environment, especially in water environments (e.g., [6,7,8,9]). Water environments are of central importance to environmental monitoring, as they serve as receiving bodies for inputs of antimicrobials, resistant microorganisms, and their antibiotic resistance genes (ARGs) from various sources, including agriculture, wastewater treatment plants (WWTPs), and industry. Additionally, aquatic environments have been hypothesized to serve as a microbial ecological incubator for the evolution of new resistant strains through horizontal gene transfer [10,11]. Some evidence indicates that aquatic environments are an exposure source, e.g., via recreation or as a drinking water source [12,13,14,15]. Recently, Liguori et al. [16] solicited expert and stakeholder input in the development of a decision tree to guide the selection of AMR monitoring targets and methods based on the monitoring objective. Four primary objectives of particular relevance to water utilities were identified: (1) wastewater-based surveillance to identify forms of AMR circulating in corresponding human populations; (2) identification of forms of AMR escaping wastewater treatment; (3) quantification of AMR removal rates achieved by different treatments; and (4) identification of hotspots for the evolution and dissemination of AMR.

Given that fecal indicator bacteria are already widely monitored as a water quality indicator and that corresponding methods have been extensively validated, one approach gaining attention is an adaptation of those methods via the addition of antibiotics to the culture media [17,18]. Recently, the World Health Organization published such a protocol, called the “Tricycle protocol” (in reference to the three domains of One Health), in which tryptone bile-glucuronide (TBX) agar is amended with cefotaxime (CTX), a broad-spectrum, third-generation cephalosporin, for the purpose of isolating putative extended-spectrum beta-lactamase (ESBL)-producing *E. coli* [18]. While most cefotaxime-resistant *E. coli* produce ESBL enzymes encoded by genes such as *blaCTX-M* [19,20,21,22], some strains overproduce chromosomal AmpC enzymes with a limited spectrum of activity. The confirmation of ESBL status therefore requires the demonstration of reduced susceptibility of a given strain in the presence of an inhibitor of beta-lactamases, such as clavulanic acid [18,23].

ESBL-producing pathogens cause a number of life-threatening infections that have limited options for treatment as they are resistant to commonly used antibiotics, including penicillins and cephalosporins [17,18]. The US Centers for Disease Control listed ESBL-producing Enterobacterales (referred to as Enterobacteriaceae prior to 2020) as a Serious Threat in 2019 [24], with a 32% increase in these organisms observed from 2019 to 2020 [25]. Infections with cefR and ESBL *E. coli* can necessitate the use of last-resort antibiotics, such as carbapenems and colistin [26]. ESBL-producing organisms have been detected with increasing prevalence across the world. The detection of these organisms in the environment has been inversely correlated with the implementation of antibiotic stewardship in both food-animal and human settings [17,26,27,28].

While the general advantage of culture-based methods is that they indicate a viable endpoint, a limitation of the WHO Tricycle protocol is its singular focus on *E. coli*. Other targets have been suggested for inclusion, such as ESBL Enterobacterales, *Enterococcus* spp., *Pseudomonas*, *Aeromonas*, or *Acinetobacter* [16,17,29,30,31]. Still, no single or combined set of culture-based targets will be able to fully capture all resistant bacteria in a given water sample. Molecular methods, e.g., quantitative polymerase chain reaction (qPCR), present an alternative to culture by facilitating the direct enumeration of ARGs across the microbial community. The broad dynamic range of qPCR and low detection limit can hypothetically support the calculation of removal rates and quantitative modeling efforts.

A comprehensive literature review of the application of qPCR for the purpose of AMR monitoring of wastewater, recycled water, and surface water revealed that *sul*1 and *int*I1 were the two most commonly applied gene targets [30]. *Sul*1 encodes resistance to sulfonamides [32], which were the first antibiotics to be mass-produced for use in human medicine, and resistance is correspondingly widespread. *Sul*1 measurements in surface waters have been found to be highly correlated to inputs from WWTPs and upstream agricultural operations [33,34,35,36]. *Int*I1 encodes the integrase gene of class 1 integrons, which are mobile genetic elements that often carry multiple ARGs in their cassettes. These *int*I1 genes have been correspondingly implicated in the widespread evolution and dissemination of antibiotic resistance [37]. Like *sul*1, *int*I1 is an indicator of anthropogenic pollution in the environment [35,38] and also often carries genes that help microbes to survive various environmental stressors [39]. Both *sul*1 and *int*I1 are also commonly linked to fecal pollution [40,41].

The objective of this study was to compare and contrast the trends observed and conclusions drawn when *E. coli* were enumerated on media containing cefotaxime (herein referred to as “cefR *E. coli*”, in accordance with the WHO Tricycle protocol) versus when *sul*1 or *int*I1 were quantified by qPCR across different water matrices. Specifically, the methods were compared in parallel by two laboratories across wastewater, recycled water, and surface water samples collected from six water utilities across the U.S. The findings can help to inform efforts to identify coordinated AMR surveillance efforts.

## 2. Results

### 2.1. Comparison of AMR Measures across Water Matrixes, Geographic Locations, and Water Utilities

**Figure 1:**
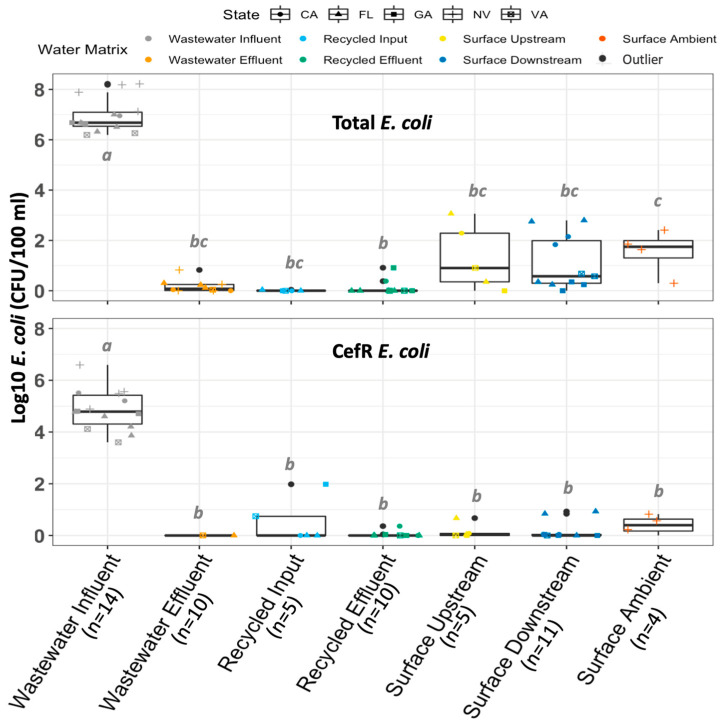
Occurrence of total *E. coli* and cefR *E. coli* cultured in this study by water matrix (indicated by color and *x*-axis). The number of samples tested is indicated in parentheses. Outliers are indicated by black data points and defined as 1.5× the interquartile range above Q3 or below Q1. Letters (a,b,c) indicate statistically significant groupings according to pairwise *t*-tests with Bonferroni correction (*p* < 0.05). Biological replicates analyzed at both Virginia Tech and the University of South Florida are combined for this analysis.

CefR *E. coli* were detected in 100% (14/14) of the influent wastewater samples, but among all other sample types tested, at least one sample yielded a non-detect. Both total *E. coli* and cefR *E. coli* concentrations differed by water matrix (Kruskal–Wallis, *p* = 5.9 × 10^−8^ and *p* = 2.2 × 10^−7^, respectively). CefR *E. coli* was most abundant in wastewater influent (Figure 1). Total *E. coli* and cefR *E. coli* measurements were very strongly correlated (r^2^ = 0.9194; *p* < 2.23 × 10^−16^).

**Figure 2:**
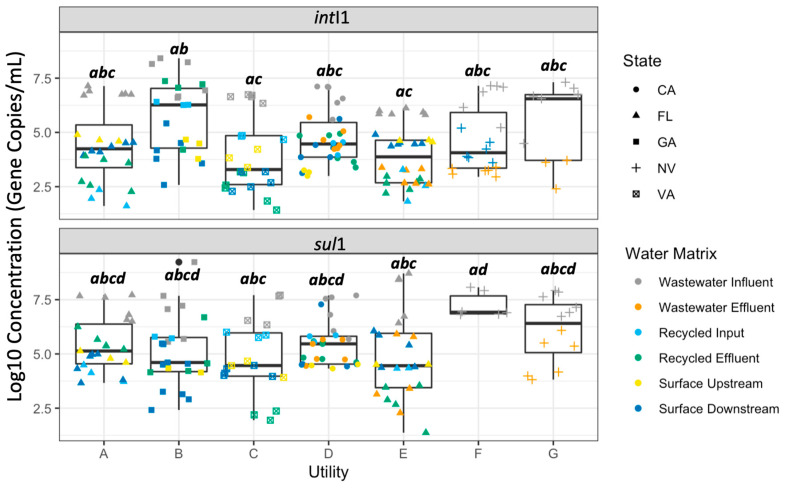
Log10 concentration (gene copies/mL) of *int*I1 and *sul*1 measured across all utilities sampled. Colors indicate the water matrix of the sample; shapes indicate the state where the utility is located. Two utilities were sampled in Nevada (NV) and Florida (FL), while only one utility was sampled in the other states. Letters indicate statistically significant groupings according to pairwise *t*-tests with Bonferroni correction (*p* < 0.05).

There were no distinct patterns observed for the *sul*1 and *int*I1 genes in the range, lowest concentration, highest concentration, or median concentration when all data points were plotted together by utility (Figure 2).

**Figure 3:**
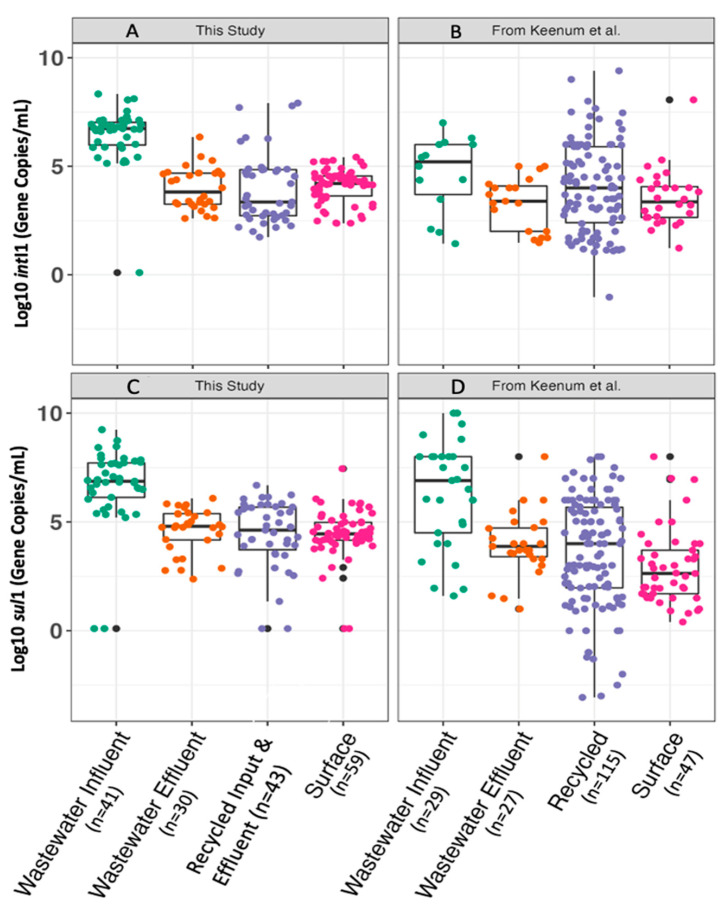
Comparison of *sul*1 concentrations in water matrices in this study (**A**) with those reported in the literature [30] (**B**); comparison of *int*I1 concentrations in water matrices in this study (**C**) with those reported in the literature [30] (**D**). Biological replicates analyzed at Virginia Tech and the University of South Florida are combined for this analysis. Data from recycled input and recycled effluent are combined under the “recycled” category in this analysis for consistency with the data available in the literature.

Figure 3 compares the gene target concentrations measured in the four categories of water matrices. The concentrations of *sul*1 that were observed here differed by type of sample (Kruskal–Wallis, *p* = 2.2 × 10^−16^). We further compared these data to those reported across 95 studies in the literature, as detailed in a meta-analysis reported by Keenum et al. [30]. The ranges and trends within and across water matrices were highly comparable between this study and that of Keenum et al. Between the two studies, neither wastewater influent concentrations of *sul*1 (*p* = 0.83) nor wastewater effluent concentrations of *sul*1 differed (Kruskal–Wallis with a Bonferroni-corrected alpha value of 0.0063, *p* = 0.030). *sul*1 concentrations measured in recycled water also did not differ between studies (Kruskal–Wallis, *p* = 0.084). The surface water concentrations of *sul*1 measured in this study were significantly higher than those reported in the literature (Kruskal–Wallis with a Bonferroni-corrected alpha value of 0.00625, *p* = 3.8 × 10^−8^).

The concentrations of *int*I1 differed by type of sample (Kruskal–Wallis, *p* = 2.2 × 10^−16^) (Figure 3). The patterns observed by water matrix in this study were also comparable to those found in the literature [30], although the *int*I1 concentration in wastewater influent and effluent exceeded the ranges found in the literature by approximately two log10. The concentrations of *int*I1 in wastewater influent were significantly higher than those reported in the literature (Kruskal–Wallis with a Bonferroni-corrected alpha value of 0.0063, *p* = 0.00024). In wastewater effluent, the concentrations of *int*I1 were not significantly different between this study and the literature (*p* = 0.10). No differences were found in terms of *int*I1 concentration in recycled water (*p* = 0.79 for *int*I1). *Int*I1 concentrations measured in surface waters in this study were significantly higher than those reported in the literature (*p* = 0.0045).

### 2.2. Correlation of sul1 and intI1 with cefR E. coli

**Figure 4:**
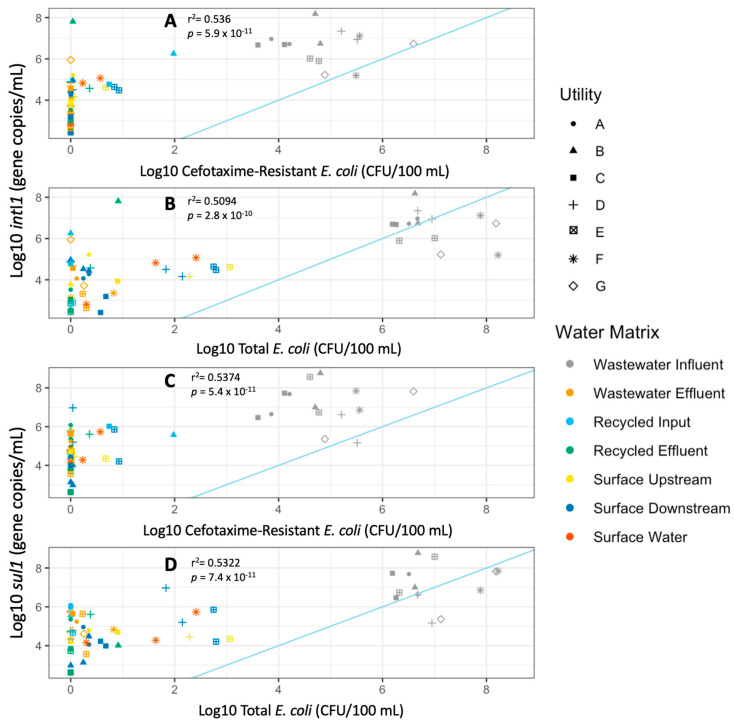
*int*I1 (**A**,**B**) and *sul*1 (**C**,**D**) are plotted against culturable cefR *E. coli* and total *E. coli*. Shapes indicate the utility where the sample was collected; colors indicate the water matrix of the sample. Biological replicates analyzed at both Virginia Tech and the University of South Florida are combined for this analysis. Light blue lines delineate a 1:1 relationship.

*Sul*1 and *int*I1 measurements were significantly correlated with cefR *E. coli* measurements across all sample types (Figure 4). However, the strengths of the correlations were moderate (r^2^~0.5). Notably, *sul*1 and *int*I1 were above the detection limit in 96.5% and 99% of the samples, respectively. On the other hand, total *E. coli* and cefR *E. coli* were above the detection limit in 76% and 50.8% of the samples, respectively. Thus, the greater number of non-detects with the culture-based analysis weakened the correlation.

*sul*1 and *int*I1 were also found to be positively correlated across all samples (Figure S2). Here, the linear regression model indicated that the correlation was statistically significant (*p* = 6.5 × 10^−8^) and moderate in strength (r^2^ = 0.40).

### 2.3. Interlaboratory Comparison

Total *E. coli* and cefR *E. coli* concentrations were very strongly correlated between the two laboratories performing the assay (r^2^ = 0.9822, *p* = 2.2 × 10^−16^; and r^2^ = 0.9783, *p* = 2.2 × 10^−16^, respectively) (Figure S3). No significant difference was found between the two labs for total *E. coli* (*p* = 0.32) or cefR *E. coli* (Kruskal–Wallis, *p* = 0.054).

The qPCR measurements of *int*I1 and *sul*1 produced by the two labs were positively correlated by linear regression (*p* = 1.1 × 10^−8^ for *int*I1 and *p* = 0.00013 for *sul*1), with weak-to-moderate correlation coefficients (r^2^ = 0.43 and 0.24, respectively) (Figure S4). No significant difference was detected between labs for *int*I1 (*p* = 0.48) or *sul*1 (*p* = 0.39) concentrations by Kruskal–Wallis. Note that samples from the recycled input and surface upstream water matrices were only analyzed at the VT lab. There is no USF comparison point, and therefore these two sample types were omitted from this analysis.

### 2.4. Repeatability of Technical and Biological Replicates

The majority of culture replicates varied less than <10% from the sample mean. Specifically, technical replicates in 121/136 total *E. coli* assays and 118/136 cefR *E. coli* assays were within 10% difference from the mean. The biological replicates in 114/130 total *E. coli* assays and 118/130 for cefR *E. coli* assays were within 10% difference from the mean (Figure S5). The water matrix (Levene Test, *p* = 0.0028) and the utility sampled (Levene Test, *p* = 6.4 × 10^−5^) had a significant effect on the percent deviation from the mean. The most variation amongst technical replicates was found for surface water samples, particularly those from utilities A and C. The variance among biological replicates deviated as much as 100% from their mean. Unlike technical replicates, there were no obvious trends in the deviation from the mean of biological replicates as a function of the water matrix. However, the utility where the sample was collected did have a significant impact on the deviation from the mean (Levene, *p* = 0.021).

Similarly to culture-based data, the variation of the replicates from the mean for qPCR data was <10% for the majority of samples (Figure S6). For technical replicates, 373/384 and 456/474 fell within 10% difference from the mean for *sul*1 and *int*I1, respectively. For biological replicates, 124/135 and 139/155 fell below this threshold for *sul*1 and *int*I1, respectively. However, the percent deviation from the mean was significantly affected by the water matrix (Levene Test, *p* = 0.0044) and the utility sampled (Levene Test, *p* = 1.2 × 10^−14^). The greatest variation among technical replicates was observed for wastewater samples. Overall, the *int*I1 assay yielded a greater percent deviation between technical replicates than the *sul*1 assay (*t*-test, *p* = 0.022). As would be expected, greater variance among biological replicates was observed relative to technical replicates. Some biological replicates varied by as much as 50%. However, there was no obvious trend in the deviation from the mean of biological replicates as a function of the water matrix (Kruskal–Wallis, *p* = 0.075), nor was there any difference in variation amongst those replicates (Levene Test, *p* = 0.62). The differences observed in percent difference from the mean across samples were also not significantly influenced by the laboratory in which they were processed (Kruskal–Wallis, *p* = 0.19). There was, however, a significant effect of water utility on the percent difference from the mean of biological replicates (Kruskal–Wallis, *p* = 0.0083) as well as the variance amongst biological replicates (Levene Test, *p* = 0.0065).

Overall, 12 outliers were detected among the biological replicates, with 1–2 outliers found amongst each utility’s sample set. In terms of the water matrix, outliers were detected in wastewater influents (n = 4), recycled water effluents (n = 4), and downstream surface waters (n = 3).

The measurements of *sul*1 and *int*I1 in trip and field blanks were found to be significantly lower than those measured in the recycled effluent samples, both when analyzed at USF (Kruskal–Wallis, *p* = 3.3 × 10^−12^) and VT (Kruskal–Wallis, *p* = 5.4 × 10^−7^).

## 3. Discussion

### 3.1. Limitations of This Study

The sampling events in this study were conducted during the COVID-19 pandemic and were therefore only made possible by the collaborative commitment of participating water utilities. Relying on multiple researchers to collect and process samples for shipping inherently increases variability in the resulting data. We sought to mitigate the introduction of such variability to the extent possible by providing highly detailed sample protocols, discussing the protocols, and answering questions through phone conversations and email. It is noted that if the protocols used here were to be widely adopted for standardized monitoring, it would be similarly important to ensure that the protocols are clear and to implement a system to ensure that they are consistently applied. Samples were collected across a relatively diverse range of waters, covering a cross-section of regions across the US. Additional sites could include other countries, especially low- and middle-income countries. A limitation to the interpretability of the data is that samples were only collected from utilities with advanced treatment that also had the facilities and human resources to collect the samples. However, assuming that there is a centralized laboratory to analyze the samples, the sampling protocol itself was developed to be as simple as possible, incorporating a pre-prepared kit. Therefore, it is expected that the approach will be adaptable to lower-resourced districts.

A recent study found that *sul*1 and *int*I1 predict a distinct subset of ARGs when compared against the shotgun metagenomic sequencing-based ARG profiling of watersheds [42]. The examination of currently available *E. coli* whole-genome sequence data indicates that 17.27% carry the *sul*1 gene [43]. *sul*1 and *int*I1 were also confirmed to be correlated, which is consistent with the understanding that *sul*1 is typically found in the *int*I1 operon [38], though the moderate correlation in this study indicates that this is not always the case and is supported by other studies that have detected moderate-to-strong correlations [44,45,46].

### 3.2. E. coli, sul1, and intI1 Measures Correlate as Indicators of Water Quality

Here we found that *sul*1 and *int*I1 were positively correlated with both *E. coli* and cefR *E. coli*. CefR *E. coli* was also very strongly correlated to total *E. coli*. *E. coli* is a well-established indicator of the efficacy of wastewater treatment and water quality. Understanding the significance of *sul*1 and *int*I1 as indicators of mobile AMR in the environment is evolving, but they have consistently been found to strongly correlate with anthropogenic inputs [35,38,39]. The overall findings are consistent with the general idea that efforts to promote overall water quality are also likely to help mitigate AMR [3]. However, it is important to recognize that not all clinically relevant AMR in the environment is fecal-associated, and therefore other targets would need to be considered if other sources are of interest [42].

### 3.3. Linking Measurements to Human Health Risk

One advantage of monitoring cefR *E. coli* is that they represent viable microorganisms belonging to a subset of the species known to contain pathogens and a form of resistance that is highly clinically relevant [14,47,48]. Thus, cefR *E. coli* is an amenable input to human health risk assessment (HHRA) models. However, not all cefR *E. coli* captured by culture are necessarily pathogens. One study examined a diverse set of sea, lake, canal, and river water samples as well as wastewater influents and effluents from municipal, hospital, and airport WWTPs using the WHO Tricycle protocol. They found that 17.1% of the isolated cefR *E. coli* (which they refer to as ESBL *E. coli*) carried virulence markers [48]. Culture may also overlook viable but nonculturable forms and thus underestimate the associated exposure risk.

### 3.4. Applying Measurements for Assessing Removal by Treatment Processes

If the objective is to measure the removal achieved by the treatment technology, then a benefit of the qPCR of *sul*1 and *int*I1 is that it provides a wide dynamic range of detection. Both qPCR methods produced a large dynamic range of quantitation, with a limit of detection at 100 gene copies/mL and an upper detection of 1.7× 10^9^ and 2.13 × 10^8^ for *sul*1 and *int*I1, respectively. The samples measured across this study fell within the range of 1.41–8.42 log gc/mL and 1.35–9.23 log gc/mL for *sul*1 and *int*I1, respectively. *E. coli* was also measured over a wide range in this study, with a lower limit of detection as low as 0.1 CFU/100 mL up to the largest detection at 3.28 × 10^8^ CFU/100 mL. However, a challenge with culture is the need to guess and establish the correct dilution range a priori, where typically it is manageable to test a range of three 10-fold dilutions. The cefR *E. coli* method dynamic range was smaller, with a lower limit of detection at 0.2 CFU/100 mL and an upper detection value at 4 log CFU/100 mL.

US CWA guidelines are already established for *E. coli*, with NPDES generally permitting an allowable geometric mean of 126 CFU/100 mL for weekly samples over the course of 30 days when the receiving body is designated for recreational uses, as the majority of receiving bodies examined here are [49]. These criteria could potentially be modified to include culturable cefR *E. coli,* but it will be important to recognize that detection frequencies will be about 1% those of total *E. coli*. Achieving non-detects of *sul*1 and *int*I1 might not be feasible from a regulatory standpoint, at least for secondary wastewater treatment. In this study, non-detects were exceedingly rare for qPCR (1 non-detect for *int*I1; 6 non-detects for *sul*1) compared to the culture method (14 non-detects for total *E. coli*; 29 non-detects for cefR *E. coli*). Among the samples where culturable cefR *E. coli* were non-detect, *sul*1 and *int*I1 concentrations remained in the 2.98–8.76 log gc/mL and 4.15–8.18 log gc/mL range, respectively. Thus, censored data tend to be less problematic from a statistical standpoint for qPCR than culture data. A low rate of non-detects in environmental samples, except for relatively pristine sites, is consistent with the literature [30,34]. Thus, one possibility for *sul*1 and *int*I1 could be to establish log removal guidelines, as has similarly been applied to the removal of viral gene copies in water reuse systems, where the WHO recommends that technologies meet a 6–7-log removal rate [50].

### 3.5. Assessing Ecological Hot Spots for AMR Selection and Evolution

One challenging monitoring need is to be able to identify a given environment or set of conditions as representing elevated “risk” for the evolution of new forms or strains of resistance. For example, pharmaceutical wastewater and affected environments are thought to pose such a risk because of the elevated selection pressure imposed by high concentrations of multiple active pharmaceutical ingredients [51,52]. For such a monitoring purpose, it is helpful to incorporate a denominator so that relative levels can be compared. In the case of cefR *E. coli*, normalizing to total *E. coli* can provide an indication of whether the resistant form survives better than the generic form when comparing a sample before and after treatment. In this study, comparing ratios of cefR *E. coli* to total *E. coli* suggested that, although cefR *E. coli* experience a net decrease through treatment, they may have a slight selective advantage, at least under some circumstances. Previous research from Bréchet et al. [53] found relative enrichment of cefR *E. coli* in comparison to total *E. coli* through the examined wastewater treatment plant.

Similarly, it is common to track ratios of *sul*1 and *int*I1 to the universal bacterial housekeeping gene, 16S rRNA, as a means to judge whether the relative proportions of microorganisms carrying these genes are increasing or decreasing [30]. Such an approach is best applied when there are multiple points of comparison through time and space. Still, if the desire is to obtain deeper ecological insight, then metagenomic measures will provide a more comprehensive picture [54], but they are more costly and require more specialized expertise. Correspondingly, there has been a convergence of recent efforts to develop tools for classifying environmental metagenomes in terms of their “resistome risk” that take into consideration the total numbers of ARGs, their clinical relevance, their presence on a mobile genetic element, and their likelihood of being carried by or transferred to a pathogen [55,56]. Such metagenomic analytical tools similarly use denominators such as annotated 16S rRNA genes or numbers of sequencing reads [54].

### 3.6. Wastewater-Based Surveillance and Epidemiology

Wastewater-based surveillance entails monitoring influent wastewater for markers of disease to inform public health efforts, while wastewater-based epidemiology takes this a step further by relating these measures to human disease [57]. Substantial infrastructure was established for this purpose across the globe as a means to track the spread of COVID-19, and there is interest in adapting this infrastructure to track AMR [5,58,59]. In the context of the present study, *sul*1 and *int*I1 alone would not be very informative for such purposes, but adapting qPCR to track emerging ARGs of concern, such as those that encode ESBLs (e.g., *bla*TEM, *bla*CTX-M), carbapenemases (*bla*NDM, *bla*VIM, *bla*OXA), colistin resistance (*mcr*), and tigecycline resistance (*tet*X), could be of value [30,60]. There are efforts to advance metagenomic-based surveillance of AMR in wastewater, which would provide the advantage of simultaneous non-targeted detection of ARGs, mobile genetic elements, and pathogens [5,58,61]. There is a substantial trade-off in terms of the cost of metagenomics; however, even with the deepest sequencing, the detection limit will be on the order of 2-log higher than that of qPCR [54].

Tracking cefR or ESBL *E. coli* could also be of value to wastewater-based surveillance and epidemiology by providing an indication of whether such infections are elevated in a community. One fundamental advantage of culture is that the isolates can be preserved for additional analysis. For example, the isolates can be subjected to phenotypic resistance profiling and compared to clinical antibiogram data to track patterns in multi-antibiotic resistance. The isolates can also be subjected to whole-genome sequencing, which can be used not only to track the sources of *E. coli* but also the molecular origin of their ARGs (e.g., movement on a plasmid). In this way, whole-genome sequencing can contribute to an ecological understanding of the drivers of resistance.

### 3.7. Practical Considerations

We adapted the WHO Tricycle protocol by using modified mTEC instead of TBX as the selective media because the former is an approved EPA standard method (Method 1603 [62]) and therefore can more readily be adopted for AMR monitoring by US water utilities. To support this approach, we previously performed a comparison of TBX versus modified mTEC medium amended with cefotaxime and found that they produced very similar results, with mTEC and incubation in a water bath at 44.5 °C providing a small but significant increase in specificity [63]. The results of the present study further demonstrate that the cefR *E. coli* methods tested here produced highly comparable results across states, water utilities, and laboratories.

The qPCR methods examined here have been widely deployed [30] but have not been standardized. We found in this study that the data generated from the *sul*1 and *int*I1 assays were significantly correlated between labs, but there was a greater discrepancy compared to the *E. coli* culture methods. Other studies have similarly reported challenges in generating repeatable qPCR measurements between labs [64,65,66] due to variability in standard curve production and challenges with reproducibility [64,67], indicating that an effort to standardize such assays would be beneficial. Transitioning to droplet digital PCR might help address some discrepancies, particularly those associated with differences in standard curves (given that no standard curve is required) [68]. We performed a ddPCR analysis of a subset of samples and found that the measurements were significantly correlated but systematically about 0.7- and 0.9-log lower than the measurements performed at VT for *sul*1 and *int*I1, respectively (Figure S7). It could not be determined if this was due to degradation of the DNA extract due to an additional freeze-thaw cycle or because the qPCR assay systematically overestimated the number of gene copies relative to ddPCR.

A challenge for all culture-based methods is that the samples are subject to short holding times and must be analyzed upon receipt within a limited time window. As a point of comparison, this study had six sample types, in both biological and technical duplicates. For this level of replication for six samples, ~5 h was required for a team of two experienced researchers on day one of processing, followed by an additional 1–2 h a day of labor for the next 2–4 days. The number of additional days needed is dependent upon how many isolation attempts are needed before reaching a pure culture, which is ultimately used for confirmation of species using PCR and/or isolated for storage [69]. Final results after species confirmation are attainable two–four days after processing.

In general, qPCR methods allow for greater flexibility in their execution because, once samples are received and concentrated, they can be preserved for extended periods at −20 °C or −80 °C prior to or after DNA extraction [70]. In this study, six samples in biological and technical triplicates (+standard curves and controls) could be analyzed for both *sul*1 and *int*I1 within 3 workdays, given two lab workers and two thermal cyclers. Day one was dedicated to filter concentration and storage of filters, day two to DNA extraction, and day three to qPCR. Thus, person-hours of labor were estimated at 20 for the culture (including time to prepare the media in advance) and at 30 for qPCR for the corresponding batches of all 72 and 48 samples, respectively. Based on our experience from this study, we estimate that as many as 8–16 samples could be analyzed for the two assays by qPCR in one batch, while 8–12 samples would be the limit for the culture (these numbers are exclusive of biological and technical replicates).

A cost analysis indicated that, for six samples collected in triplicate and run with technical duplication as in our study, the cost of consumables is between USD 120 and USD 225 for the culture method. For a batch of six samples collected in biological triplicates and analyzed in technical triplicates by qPCR, the cost of consumables is between USD 570 and USD 650 for the methods performed here (Table S4). In terms of capital costs, a culture requires an autoclave, a clean workspace, a water bath, and an incubator (Table S5). The most essential capital cost for qPCR is a real-time thermal cycler, which can cost on the order of USD 20,000-40,000, as well as a homogenizing instrument to facilitate DNA extraction. qPCR generally also requires a −20 °C freezer for storage of filters and DNA extracts if the analysis is not immediate. The conversion to droplet digital PCR incurs even greater capital costs but may be worthwhile if the results are more consistent and require less troubleshooting. Given that US utilities are already required to monitor *E. coli*, there is generally less of a cost barrier as an entry point. However, water utilities in the US are more commonly equipped with DNA analysis capabilities, including thermal cyclers and technicians trained on qPCR methods. Liguori et al. [16] found that eight out of nine utility employees surveyed reported that their organization has the capacity to run qPCR.

### 3.8. QA/QC Recommendations

An advantage of the *E. coli* method is that QA/QC criteria have already been established in a manner that harmonizes with existing fecal indicator regulatory guidelines. However, we found that for a large-scale comparative study of this nature, it was not practically feasible to process the samples within the 2–8 h window specified by EPA Standard Method 1603. We instead set a limit at 48 h to allow time for shipping and held the samples on ice to reduce die-off. The high level of replication between labs, despite this modification, is encouraging. A study that examined the effects of holding time on the survival of culturable *E. coli* found that *E. coli* concentrations in 64% of the samples that were held on ice and concentrated by membrane filtration did not change significantly after 48 h [71]. While it is desirable to process samples for culturable *E. coli* as quickly as possible, studies across large geographic areas require shipping and extended holding times, and *E. coli* concentrations are generally stable if samples are held on ice.

Borchardt et al. [72] recently recommended several QA/QC measures to improve the accuracy and repeatability of qPCR when applied to environmental samples, referred to as the EMMI guidelines. The outlined QA/QC processes were included in this study, with the exception of the 95% probability model for calculating LOD. While the qPCR measurements in this study were significantly correlated between laboratories, the correlations were weak, and there were a number of samples with fairly discrepant measures. Efficiencies and r^2^ coefficients of the standard curves were found to differ between the laboratories, which might have contributed to the observed variability. Standard curves were included in each run, as recommended by EMMI, but errors in the standard preparation/quantification or differences in the sample and standard chemistry could still affect the measurements. Some sample types also have inherently higher variability among biological replicates, which could contribute to these differences. For example, grab samples collected under plug-flow conditions in the WWTP or in corresponding surface waters will be subject to random differences. However, significant differences in the variance among biological qPCR replicates were only noted as a function of the water utility, not the water matrix. Such variance as a function of the water utility could be a result of different personnel collecting the water or unique on-site challenges to homogeneous sample collection. Interestingly, both the sample matrix and the utility also had a significant impact on variance among technical replicates. This suggests that water chemistry might influence the DNA extract chemistry and correspondingly influence measurements, e.g., through PCR inhibition.

In a power analysis for linear regression determined for six water matrices, two labs, and six utilities (72 qPCR samples), a sub-group sample size of 2.77 is required to obtain significance at the level of 0.05 and a power of 0.8. This analysis indicates that, at a minimum, biological triplicates are necessary to support statistical comparison for qPCR analysis. It was also noted that the *sul*1 assay produced less variance among technical replicates as compared to the *int*I1 assay (Figure S6. This *sul*1 assay in particular has been used historically in both laboratories and is well established, which could account for some improved consistency across replicates.

In a power analysis for linear regression determined for four water matrices, two labs, and six utilities (48 culture samples), a sub-group sample size of 1.5 is required to obtain significance at the level of 0.05 and a power of 0.7. This indicates that, at a minimum, biological duplicates are necessary to support statistical comparison for culture analysis. For total *E. coli* and cefR *E. coli*, technical replicates centered closely around their mean, but variation from the mean did ultimately differ significantly by utility and water matrix. Some variation amongst technical replicates could be explained by consistency in the researchers’ technique, and improved homogenization of the sample and matrix prior to filtration may be able to reduce this effect [73,74]. The biological replicates also varied by utility and water matrix, with a larger scale of variation observed upwards of 1-log for both total *E. coli* and for cefR *E. coli*. The greatest variance within each sample type was noted for wastewater influent and effluent, which is reasonable given the inherent heterogeneity observed as plugs of wastewater flow through a treatment plant. A fair degree of variance was also observed across the surface water samples included in this study, indicating that some variation amongst the biological replicates was contributed by local factors, which include the chemistry of the wastewater or the differences in the technique applied by the personnel collecting the samples. Surface water is also subject to mixing and flow conditions that can contribute to the variance in the biological replicate samples.

## 4. Materials and Methods

### 4.1. Sampling and Interlaboratory Comparison

Samples for this study were collected from six utilities, each incorporating both wastewater and water reuse treatment trains, located in five different US states, over a six-month period. Two utilities were sampled in Florida and Nevada, while one utility was sampled in each of the other states. We worked with each utility to develop and implement a tailored sampling plan that captured influent and effluent to the WWTP, input and effluent to water reuse treatment trains, and a nearby surface water body, ideally one receiving WWTP effluent if possible. For utilities with surface-water discharge sites, samples were collected both upstream and downstream of the discharge pipe, with locations specific to each site due to feasibility and accessibility. A detailed sampling protocol was developed and is reported in Liguori et al. 2023 [75]. Briefly, each utility partner was provided with sampling kits, which included all supplies, protocols, and return shipping labels. Sampling kits were assembled identically for each sample event, with the number and labeling of bottle sets being adapted for each utility in order to simplify sample collection for utility personnel and avoid human error such as mislabeling/misinterpretation of sample kit contents. Duplicate kits were provided for parallel sample collection and shipment for comparative analysis in the laboratories at Virginia Tech (VT) and the University of South Florida (USF). All sample types were collected and analyzed in parallel, with the exception of recycled water input and surface water upstream samples, which were only analyzed at VT. Throughout the sampling, processing, and analysis steps, Quality Assurance and Quality Control (QA/QC) were prioritized. Full details about the quality assurance protocol can be found in [75].

WWTP influent samples (50 mL) were collected after the grit chamber but before primary settling. Wastewater effluent samples (2 L) were collected following secondary clarification and not following any further tertiary treatment for uniformity of comparison across plants. Recycled water samples (2 L) were collected to capture the water entering recycled treatment (“input”, typically secondary effluent or tertiary treated water piped to the recycled treatment facility) and the final treated recycled water effluent coming out of the facility. Samplers wore clean gloves, and samples were collected in sterilized bottles. Sample valves/ports were flushed for 30 s before the respective sample bottle was uncapped and filled to the bottom of the bottle stem. Bottles were then recapped and placed into the cooler with ice packs.

Surface water samples (2 L) were collected upstream and downstream of the wastewater discharge point to the water body, where possible. The sampling bottles were placed about one foot below the water surface, with the cap still on, upstream of the individual collecting the sample. Once below the water surface, the cap was removed and the bottle was filled, then recapped before removing it from the water. This process was repeated for each surface water bottle at their respective locations in the surface water body. All bottles were maintained on ice for preservation and transport.

A trip blank consisted of 1 L of molecular-grade or autoclaved water that was shipped with the sampling kits, remained closed throughout the sampling, and was returned with the bottles. A field blank consisted of 1 L of the same water, but the bottle was opened during sampling to capture potential contamination resulting from field sampling.

All samples were shipped overnight to enable processing at the laboratories within 24 h of sampling. Upon receipt, all samples were maintained on ice or refrigerated until processed, up to 8 h. Processing included filter concentration with nitrocellulose or mixed-cellulose ester filters for both culture and molecular qPCR methods, using filter sizes of 0.45 µm and 0.22 µm, respectively. Small volumes (under 5 mL) were suspended in sterile buffer prior to application to the filter cups, while sample volumes of 5 mL or greater were added directly to the filtration cups. Detailed analytical standard operating procedures developed and used by both laboratories are available at https://ward.cs.vt.edu (accessed on 30 June 2023) and Liguori et al. [75] and summarized in Table 1.

### 4.2. CefR- E. coli Culture

All samples were cultured on modified mTEC media (BD Difco Cat No. B14880) and modified mTEC with 4 µg/mL cefotaxime (Millipore Sigma Product No. 219504; Fisher Sci Cat No. AC454950010). The methods used herein were adapted from EPA Method 1603 for *E. coli* [62] and the WHO Global Tricycle Surveillance of ESBL-*E. coli* method [18]. At each lab and for each sample, replication was achieved both in biological duplicates (i.e., separate sampling bottles) and technical duplicates (i.e., separate culture plates). From each biological duplicate bottle, 3 target dilutions were filtered onto membranes in technical duplicate (~24 plates were typically analyzed per sample (2 biological duplicates × 3 dilutions × 2 technical duplicates × 2 media types)).

Serial dilutions of wastewater influent samples were made prior to plating in order to capture a countable range of colonies. To achieve these dilutions, 1 mL of sample was added to a tube containing 9 mL of sterile phosphate-buffered saline (PBS). This was repeated serially to achieve a final dilution of 10-4. For all other sample types, the original sample was added to the filter concentration setup without dilution. The volume of sample filtered differed based on sample type, as outlined in Table S1.

Samples were concentrated by filtration onto 0.45 µm filters with a 47 mm diameter (nitrocellulose: Fisher Cat No. 09-719-555; mixed-cellulose ester: Fisher Cat No. HAWP04700) and plated in a biosafety level II cabinet. Flame-sterilized forceps were used to place filters inside autoclaved filter cups. The appropriate volume for each sample type (Table S1) was then added to the filter cup, which was capped before turning on the vacuum filter and allowing the sample to pass completely through the filter into the receiving cup. For volumes of 1 mL, the samples were suspended in 30 mL of sterile PBS prior to filtration to improve spread across the filter. After the vacuum was turned off, the filter was removed using flame-sterilized forceps and placed onto the respective media plate. This process was repeated for all samples, including negative and positive controls, from “cleanest” to “dirtiest”, utilizing previous experience of working with these sample types to estimate which samples were likely to contain the least/most number of culturable *E. coli* to determine their order, with each cup being rinsed with sterile PBS in between samples. Plates were inverted and transferred to an incubator at 35 °C for two hours. After 2 hours, these plates were transferred to a pre-heated water bath at 44.5 °C for 22 h (± 2 h). Setup and submersion of plates in the water bath are detailed further in the Supplemental Information (Figure S1).

After the 20–24 h incubation, plates were removed from the water bath, and colony-forming units (CFUs) were enumerated on all plates, manually marking each colony with a permanent marker. Numbers are reported here for dilutions that produced CFUs in a countable range (10–100 CFU per plate). Species confirmation was conducted on five colonies per water matrix, per sampling event, up to a total of 30 samples per sampling event where sufficient colonies grew (Table S2).

### 4.3. qPCR Enumeration of sul1 and intI1

All samples designated for qPCR analysis were filtered to clogging onto 0.22 µm filters with a 47 mm diameter (Millipore Sigma Cat No: GSWG047S6). Clogging was defined as no liquid penetrating the filter for a period of 10 or more minutes. All sample bottles were weighed before and after filtering to determine the net difference and, therefore, estimate the volume of sample filtered. Autoclaved filter cups and flame-sterilized forceps were used in a biosafety cabinet to minimize potential for sample contamination.

Filters were removed from the cups using flame-sterilized forceps and folded into sterile O-ring microcentrifuge tubes for storage. A filter blank (50 mL of sterile PBS) was filtered at each sample processing event to serve as a negative control throughout the extraction process. Each tube containing a filter was filled to cover the filter with a 50% ethanol solution to prevent degradation of DNA in storage and stored at −20 °C until DNA extraction within 20 days [74]. After thawing at room temperature, filters were removed and shredded using flame-sterilized forceps and subsequently extracted using the FastDNA SpinKit for Soil (MP Bio Cat No. 6560200). When the SpinKit directions suggested a range of options, the more conservative (e.g., longer time, high temp., larger tube) was implemented. The resulting DNA extracts were stored in molecular-grade water at −20 °C.

qPCR was performed in technical triplicate using primers and probes, where applicable, as reported previously by Pei et al. [33] and Barraud et al. [76] for *sul*1 and *int*I1, respectively (Table 1). For the qPCR master mix, 12.5 µL of PowerSYBR Green was used for dye assays and TaqMan Environmental Master Mix 2.0 for probe-based assays, with a total reaction volume of 25 µL per well. Standard curves were created using Gblocks (Integrated DNA Technologies, Coralville, IA, USA) designed specifically for each gene target (sequence available in Table S3). Gblocks were serially diluted and analyzed along the first row of each qPCR plate for each standard curve, with the typical dilution ranging from 108 to 102. All qPCR analysis was conducted on a Bio-Rad CFX RT-PCR thermal cycler (Bio-Rad, Hercules, CA, USA). The resulting data were compared to those extracted from the study carried out by Keenum et al. [30] for comparable samples.

### 4.4. Statistical Analysis

The dilution that produced CFUs most centrally within the countable range was reported for culture-based analysis. The CFUs/mL for the selected dilution were averaged across the two technical replicates (A and B) and converted to a per 100 mL basis to produce the final count of CFUs/100 mL for each sample. The limit of detection was identified for each plate as equivalent to 1 colony per the highest volume of sample plated. Non-detects were assigned a value of one-half the limit of detection. For lawns and growth that were too numerous to count, a value of 750 was assigned as an arbitrary high value > highest countable number. Culture data were compiled and averaged in Excel, then loaded into R (version 4.1.2) for all statistical analyses.

qPCR data were extracted from Bio-Rad CFX manager instrument software (version 3.1). The lowest standard that produced quantifiable concentrations in 2/3 of the technical replicates was set as the limit of detection. The lowest standard that produced quantifiable concentrations for 3/3 of the standard technical replicates was set as the limit of quantification. Technical replicates were averaged for all samples for which at least 2/3 of the technical replicates produced a measurable gene target. Samples with a signal below the limit of detection were marked as non-detects and assigned a value of 1.25 gene copies per milliliter for statistical purposes. Measurements falling between the limit of detection and limit of quantification were assigned a value half that of the limit of quantification. The qPCR data were compiled in Excel and transferred into R (version 4.1.2) for all statistical analyses.

All culture and qPCR data were log-transformed prior to analysis. Neither culture nor qPCR data were found to be normally distributed according to the Shapiro–Wilks test. Kruskal–Wallis was used as a non-parametric test for differences between groups. Levene tests were used to test for differences in variance between groups. Linear regressions were used to assess the strength of correlations (r-squared values) across all water matrices. Additionally, Spearman’s rank correlations were used to assess the strength of correlations within each water matrix to separate effects from water type (Table S6). Statistical significance was set across this study at *p* < 0.05. Bonferroni adjustments to p-values were made where applicable by dividing by the number of simultaneous tests being performed.

To assess the regression coefficient between culture data and qPCR data on a sample-by-sample basis, the biological triplicates from qPCR were averaged in order to produce a dataset with even numbers of samples in each group (qPCR and culture). In order to assess replication, technical and biological replicates were averaged, and then each sample was compared to its corresponding average. To determine the percent deviation from the mean, the mean was subtracted from each of the individual replicate concentrations, then that was divided by the mean and multiplied by 100. Outlier analysis was conducted utilizing the interquartile ranges calculated in the geom_boxplot function of ggplot2 with outlier detection enabled.

## 5. Conclusions

This study provides a systematic comparison of candidate targets for global monitoring of AMR in water environments. Data were compared from six distinct wastewaters, recycled waters, and surface waters across the US. The general trends produced by cefR *E. coli*, *sul*1, and *int*I1 enumeration were similar, i.e., highest in raw wastewater and lowest in recycled water effluents, and all three measures were significantly correlated with each other. Although the intention for the selection of the targets in this study was for them to serve as indicators of AMR, it is important to recognize that they measure distinct aspects of AMR and therefore have their own limitations in terms of technical and logistical significance. Therefore, there are a number of considerations in selecting a target for AMR monitoring, depending on the monitoring objective and the circumstances [8,16]. This study provides detailed practical guidance for implementing these targets for the purpose of AMR monitoring in aquatic environments, including considerations with respect to the resources required, repeatability, comparability, ease of implementation, QA/QC, and the overall monitoring objectives that they are suitable to address. Additional work is needed to test and compare alternative culture targets, such as *Enterococcus*, and qPCR targets, such as *bla*CTXM, *van*A, and *tet*A.

## Figures and Tables

**Table 1:** Methods Compared in this Study.

Method	Target	Rationale	Details	Assay Reference
Culture	Total *E. coli*	Comparison point for cefR *E. coli* and allows percent resistance calculations	Modified mTEC	EPA Method 1603 [62]
	cefR *E. coli*	Can cause difficult-to-treat resistant infections	Modified mTEC with cefotaxime	Modified from EPA Method 1603 [62]
qPCR	*int*I1	Indicator of mobile, anthropogenic sources of multi-antibiotic resistance	TaqMan probe assay (5′-3′): F: GCCTTGATGTTACCCGAGAG; R: GATCGGTCGAATGCGTGT; P: (6-FAM) ATTCCTGGCCGTGGTTCTGGGTTTT (BHQ1)	Barraud et al., 2010 [76]
	*sul*1	ARG that encodes resistance to sulfonamides and correlates with anthropogenic inputs	F (5′-3′): CGCACCGGAAACATCGCTGCAC; R (5′-3′): TGAAGTTCCGCCGCAAGGCTCG	Pei et al., 2006 [33]

## Data Availability

The data, protocols, and QA/QC procedures produced in this study are available in the Water Research Foundation report for Project #5052 published 20 January 2023, which can be found at https://www.waterrf.org/resource/standardizing-methods-qaqc-standards-investigating-occurrence-and-removal-antibiotic-3.

## Supplementary Materials

The following supporting information can be downloaded at: https://www.mdpi.com/article/10.3390/antibiotics12081252/s1, Figure S1: Setup and submersion of culture plates in the water bath; Figure S2: Correlation of *sul*1 and *int*I1 (gene copies/mL), with shape indicating utility where sample was collected and color indicating water matrix. Adjusted linear regression r-squared value and *p*-value are reported. Biological replicates analyzed at both Virginia Tech and the University of South Florida are combined for this analysis. Light blue lines delineate a 1:1 relationship. Figure S3: Interlab comparison of total *E. coli* (A) and cefR *E. coli* (B) concentrations measured across water matrices, with USF on the x-axis and VT on the y-axis. Note that recycled input and surface upstream samples were not collected at USF and therefore were omitted in this analysis. Light blue lines delineate a 1:1 relationship. Figure S4: Interlab comparison of *intI*1 (A) and *sul*1 (B) qPCR measurements. Recycled input and surface upstream samples were not analyzed at USF and are excluded from this analysis. Light blue lines delineate a 1:1 relationship. Figure S5: The percent difference from the mean for each technical replicate for total *E. coli* (A) and cefR E. coli (B) and biological replicate for total *E. coli* (C) and cefR *E. coli* (D) of each sample cultured (samples are unlabeled across the X-axis). Colors indicate water matrix sample; Shapes indicate utility where sample was collected. The mean here is the average of three technical and biological replicates, respectively, collected at the same site and time, and processed at the same laboratory. The horizontal line indicates 0% deviation from the mean as a reference. Replicates analyzed at both Virginia Tech and University of South Florida are all included in this analysis. Figure S6: The percent difference from the mean for each technical replicate for *int*I1 (A) and *sul*1 (B) and biological replicate for *int*I1 (C) and *sul*1 (D) of each sample (samples are unlabeled across the x-axis). Colors indicate water matrix sample; shapes indicate utility where sample was collected. The mean here is the average of three technical and biological replicates collected at the same site and time and processed at the same laboratory. The horizontal line indicates 0% deviation from the mean as a reference. Replicates analyzed at both Virginia Tech and the University of South Florida are all included in this analysis. Figure S7: Correlation of *sul*1 (A) and *int*1 (B) concentrations (gene copies/ml) by qPCR and ddPCR. Color indicates water matrix. Adjusted linear regression r-squared value and p-value are reported. Biological replicates analyzed at Virginia Tech are included in this analysis. ddPCR was conducted by partners at US EPA’s Office of Research and Development. Light blue lines delineate a 1:1 relationship. Table S1: Sample volumes used to concentrate samples for culture assays by membrane filtration; Table S2: PCR Sequence for species confirmation by *uid*A; Table S3: qPCR Sequence *sul*1/*int*I Gblock standard. Table S4: Estimates and assumptions for cost analysis of methods for 6 samples (plus biological and technical triplicates for qPCR and biological triplicates and technical duplicates for culture). Table S5: Capital Costs Associated with Methods; Table S6: Spearman’s correlation for each set of variables for which overall correlations were presented in this paper, with relationships tested within each individual water matrix to assess impact of water matrix variety. Reference [69] is cited in the Supplementary Materials.
