# Supplementary material for: Comparison of Cefotaxime-Resistant Escherichia coli and sul1 and intI1 by qPCR for Monitoring of Antibiotic Resistance of Wastewater, Surface Water, and Recycled Water
Source: Antibiotics (Basel). 2023 Jul 29;12(8):1252. doi: 10.3390/antibiotics12081252 (PMC10451376; doi:10.3390/antibiotics12081252)
Supplement: Supplementary file 1 [file antibiotics-12-01252-s001.zip › antibiotics-2509813-supplementary.pdf]

SUPPLEMENTARY MATERIALS

Comparison of Cefotaxime-Resistant *Escherichia coli* and *sul*1 and *int*I1 by qPCR for Monitoring of Antibiotic Resistance of Wastewater, Surface Water, and Recycled Water

Authors: Krista Liguori[1]; Jeanette Calarco[2]; Gabriel Maldonado Rivera[1]; Anna Kurowski[1]; Ishi Keenum[1]; Benjamin Davis[1]; Valerie J. Harwood[2]; Amy Pruden[1]*

1 Via Department of Civil and Environmental Engineering, Virginia Tech, Blacksburg, Virginia 24060 USA

2 Department of Integrative Biology, University of South Florida, Tampa, Florida 33620 USA

*Corresponding Author

Summary of contents:

- 11 pages, 7 figures, 5 tables

Table S1: *Sample volumes used to concentrate samples for culture assays by membrane filtration*

Water Matrix	Media	Filtration Amount
Wastewater influent	Modified mTEC	1 mL (at 10^{-2},$^{-3}$,$^{-4}$)
	Modified mTEC w/ cefotaxime	1 mL (at 10^{-1},$^{-2}$,$^{-3}$)
Wastewater effluent	Modified mTEC	Up to 500 mL (until filter refusal)
	Modified mTEC w/ cefotaxime	Up to 500 mL (until filter refusal)
Recycled Water	Modified mTEC	Up to 500 mL (until filter refusal)
	Modified mTEC w/ cefotaxime	Up to 500 mL (until filter refusal)
Surface Water	Modified mTEC	1, 10, 100 mL
	Modified mTEC w/ cefotaxime	10, 100, 250 mL

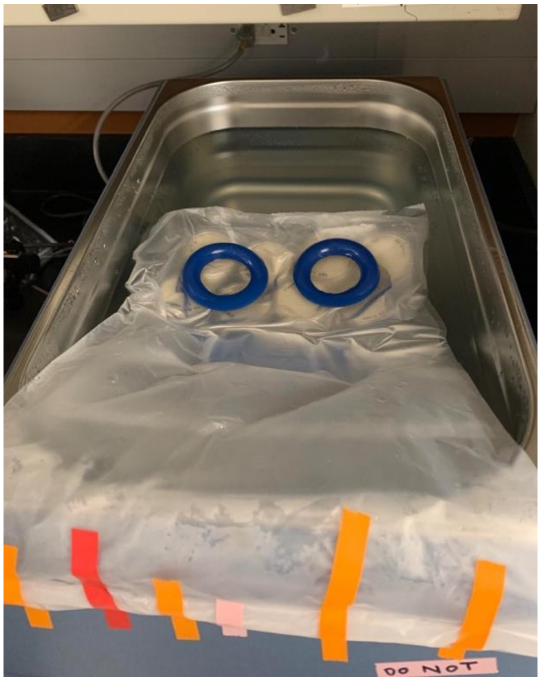

Figure S1: *Set up and submersion of culture plates in the water bath*

Table S2: PCR Sequence for species confirmation by *uid*A

Target	Type	Sequence (5' to 3')	Rationale	Reference
uidA	Intercalating dye	F) CAACGAACTGAACTGGCAGA; R) CATTACGCTGCGATGGAT	*E. coli species confirmation*	*Modified from Chern et al., 2009**

**Chern et al. assay was published using a probe and was adapted here as an intercalating dye-based assay for presence/absence of E. coli species.*

Table S3: qPCR Sequence *sul*1/*int*I Gblock standard

**Combined Gblock sequence for both sul1 and intI1:**
tgcatgatctacgtgcgtcacatgcagtacCGCACCGGAAACATCGCTGCACGTGCTGTCGAACCTTCAAAAGCTGAAGTC<br>GGCGTTGGGGCTTCCGCTATTGGTCTCGGTGTCGCGGAAATCCTTCTTGGGCGCCACCGTTGGCCTTCCTGTAA<br>AGGATCTGGGTCCAGCGAGCCTTGCGGCGGAACTTCAActgtgcggactctaCTGGATTTCGATCACGGCACGATCA<br>TCGTGCGGGAGGGCAAGGGCTCCAAGGATCGGGCCTTGATGTTACCCGAGAGCTTGGCACCCAGCCTGCGCG<br>AGCAGCTGTCGCGTGCACGGGCATGGTGGCTGAAGGACCAGGCCGAGGGCCGCAGCGGCGTTGCGCTTCCCG<br>ACGCCCTTGAGCGGAAGTATCCGCGCGCCGGGCATTCCTGGCCGTGGTTCTGGGTTTTTGCGCAGCACACGCAT<br>TCGACCGATCCACGGAGCGGTGTCGTGCGTCGCCATCACATGTATGACCAGACCTTTCAGCGCGCCTTCAAACG<br>TGCCGTAGAACAAGCAGGCATCACGAAGCCCGCCACACCGCACACCCTCCGCCACTCGTTCGCGACGGCCTTGC<br>TCCGCAGCGGTTACGACATTCGAACCGTGCAGGATCTGCTCGGCCATTCCGACGTCTCTACGACGATGATTTAC<br>ACGCATGTcactagctcagattcagtagaccgctgttg
Key:<br>Spacers<br>Sul1 Forward primer<br>Sul1 Reverse Primer Complement<br>IntI1 Forward Primer<br>IntI1 Probe<br>IntI1 Reverse Primer Complement

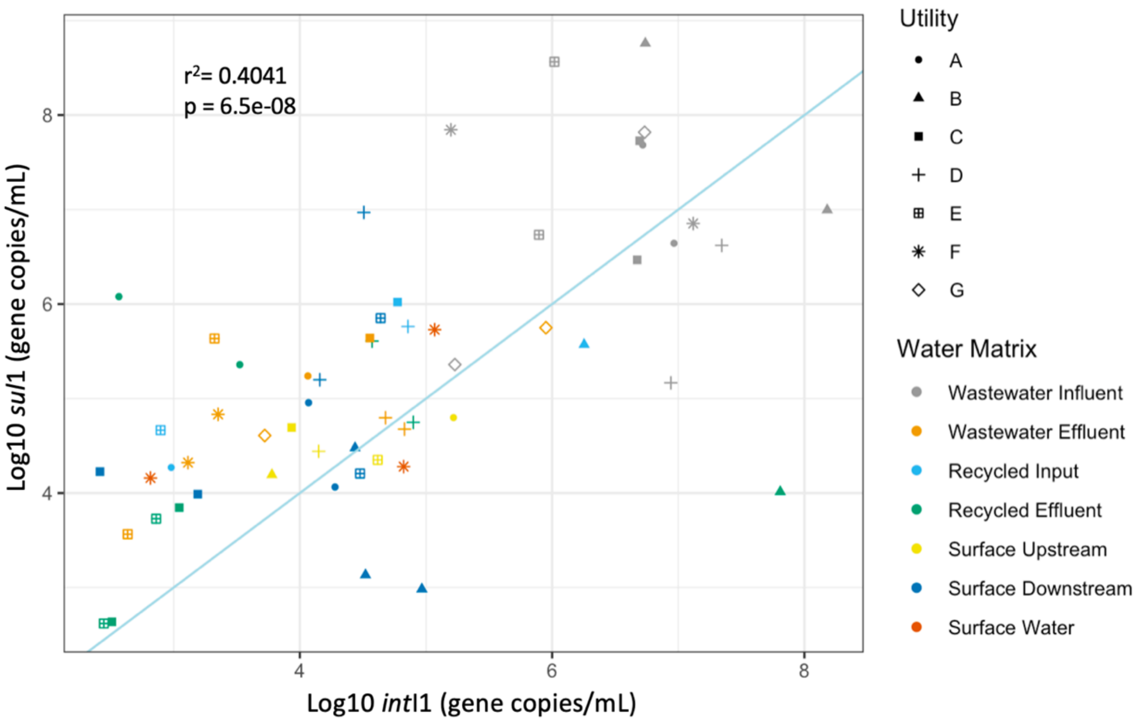

Figure S2: *Correlation of sul1 and intI1 (gene copies/ml), with shape indicating utility where sample was collected and color indicating water matrix. Adjusted linear regression r-squared value and p-value are reported. Biological replicates analyzed at both Virginia Tech and University of South Florida are combined for this analysis. Light blue lines delineate a 1:1 relationship.*

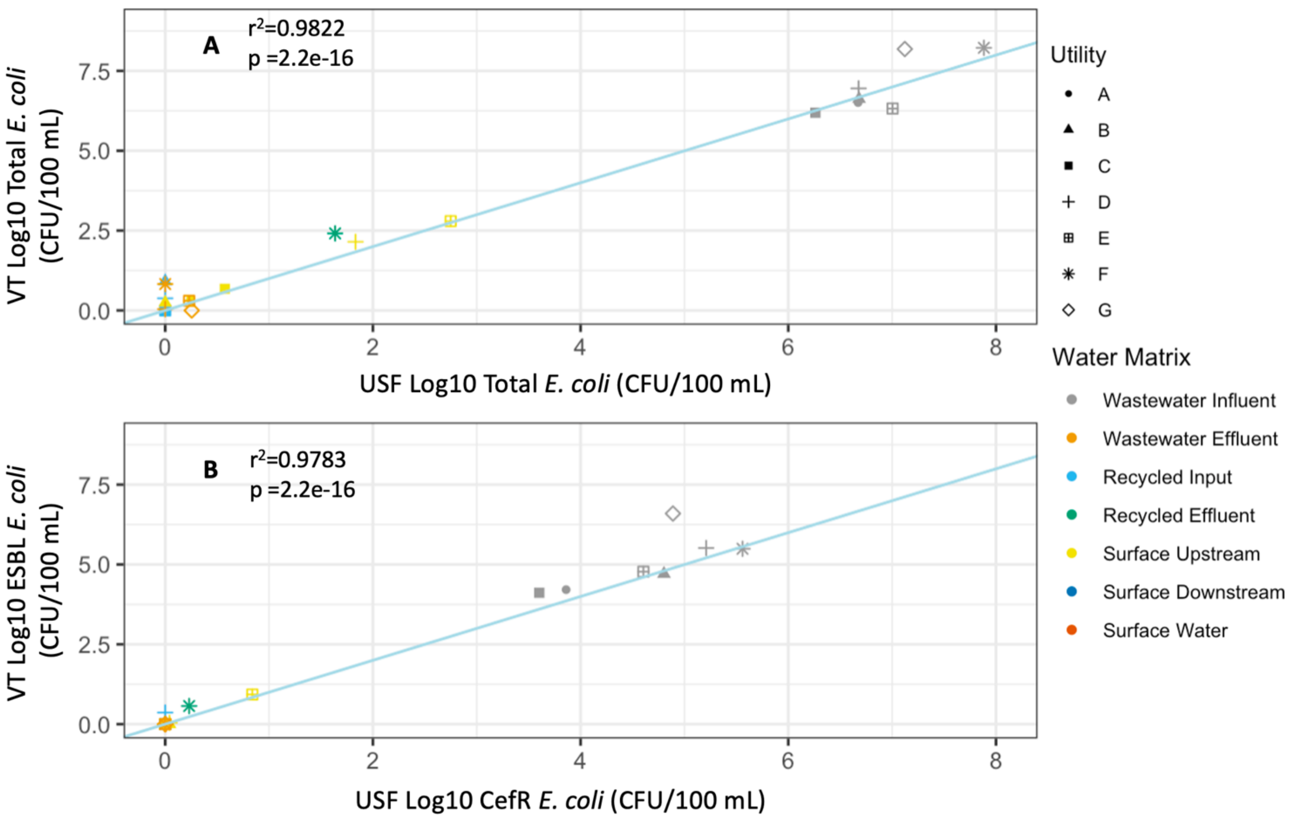

Figure S3: *Inter-lab comparison of total E. coli* (A) *and cefR E. coli* (B) *concentrations measured across water matrices, with USF on the x-axis and VT on the y-axis. Note that recycled input and surface upstream samples were not collected at USF and therefore were omitted in this analysis. Light blue lines delineate a 1:1 relationship.*

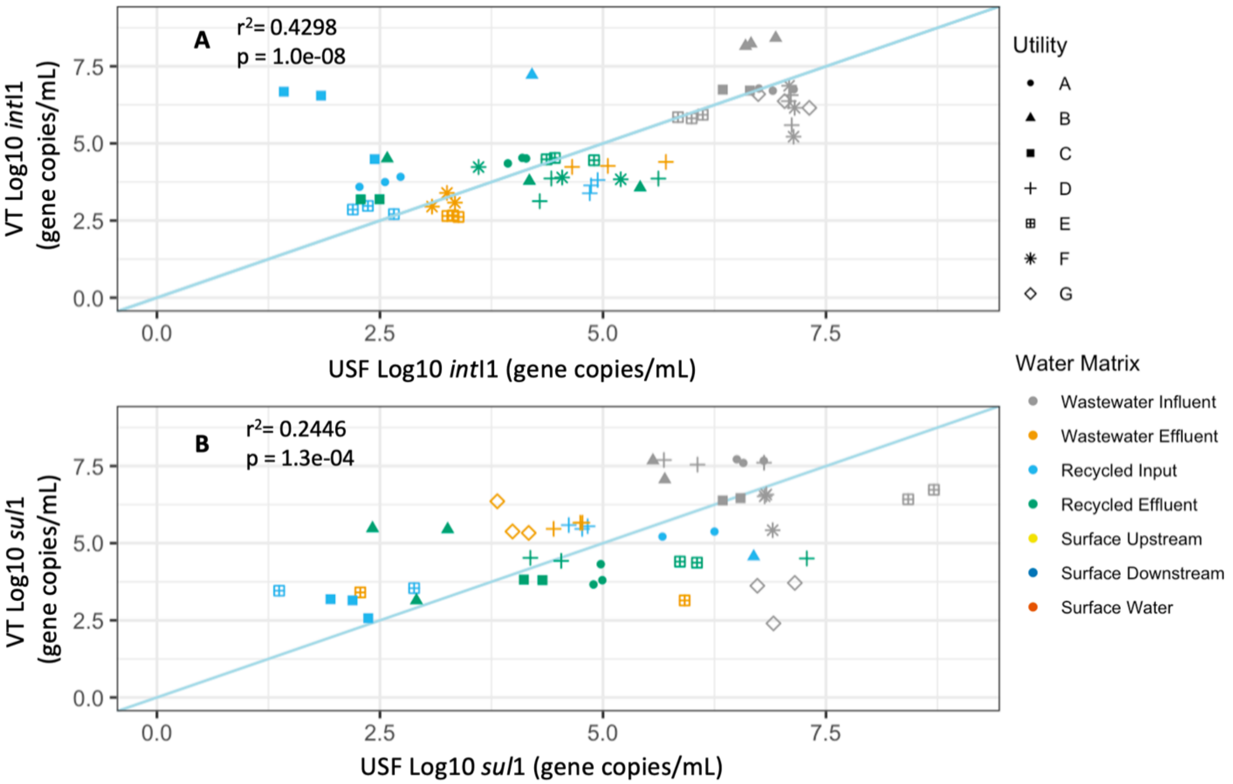

Figure S4: *Inter-lab comparison of intI1 (A) and sul1 (B) qPCR measurements. Recycled input and surface upstream samples were not analyzed at USF and are excluded from this analysis. Light blue lines delineate a 1:1 relationship.*

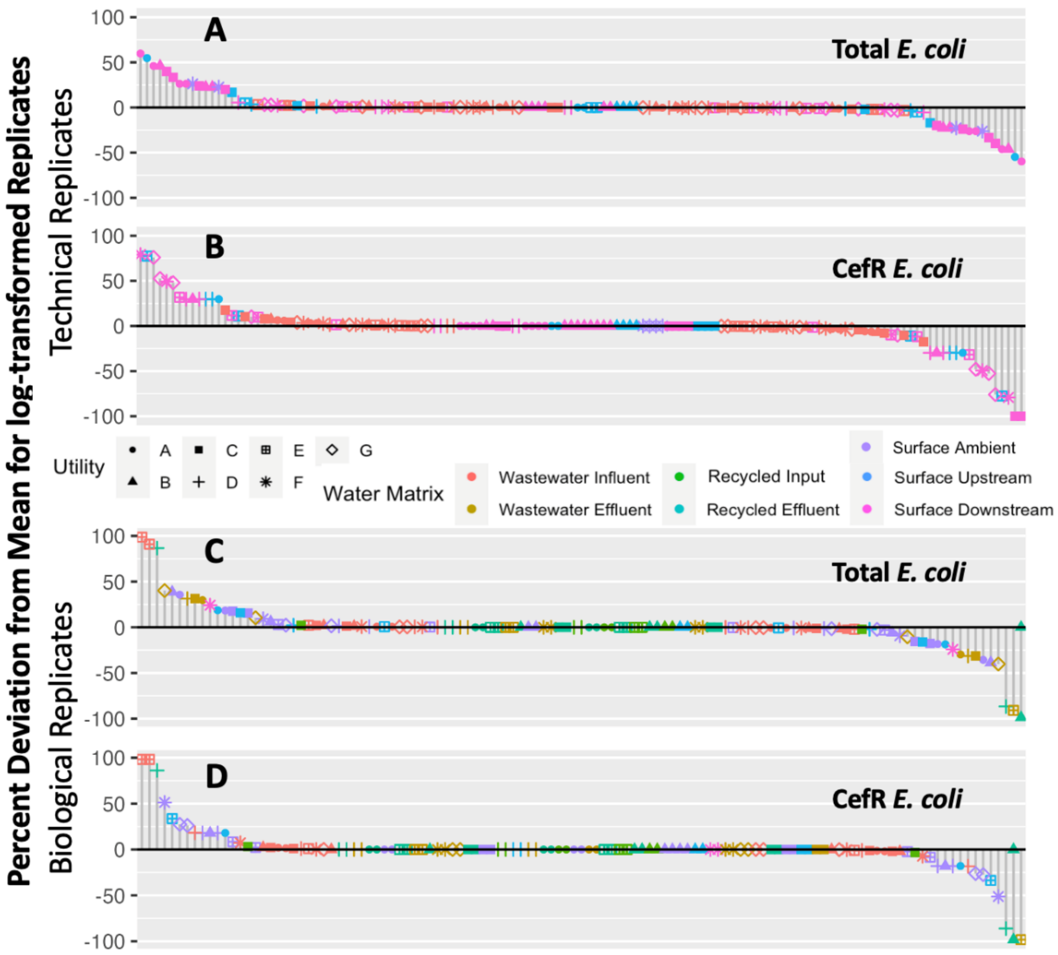

Figure S5: *The percent difference from the mean for each technical replicate for total E. coli (A) and cefR E. coli (B) and biological replicate for total E. coli (C) and cefR E. coli (D) of each sample cultured (samples are unlabeled across the X-axis). Colors indicate water matrix sample; Shapes indicate utility where sample was collected. The mean here is the average of three technical and biological replicates, respectively, collected at the same site and time, and processed at the same laboratory. The horizontal line indicates 0% deviation from the mean as a reference. Replicates analyzed at both Virginia Tech and University of South Florida are all included in this analysis.*

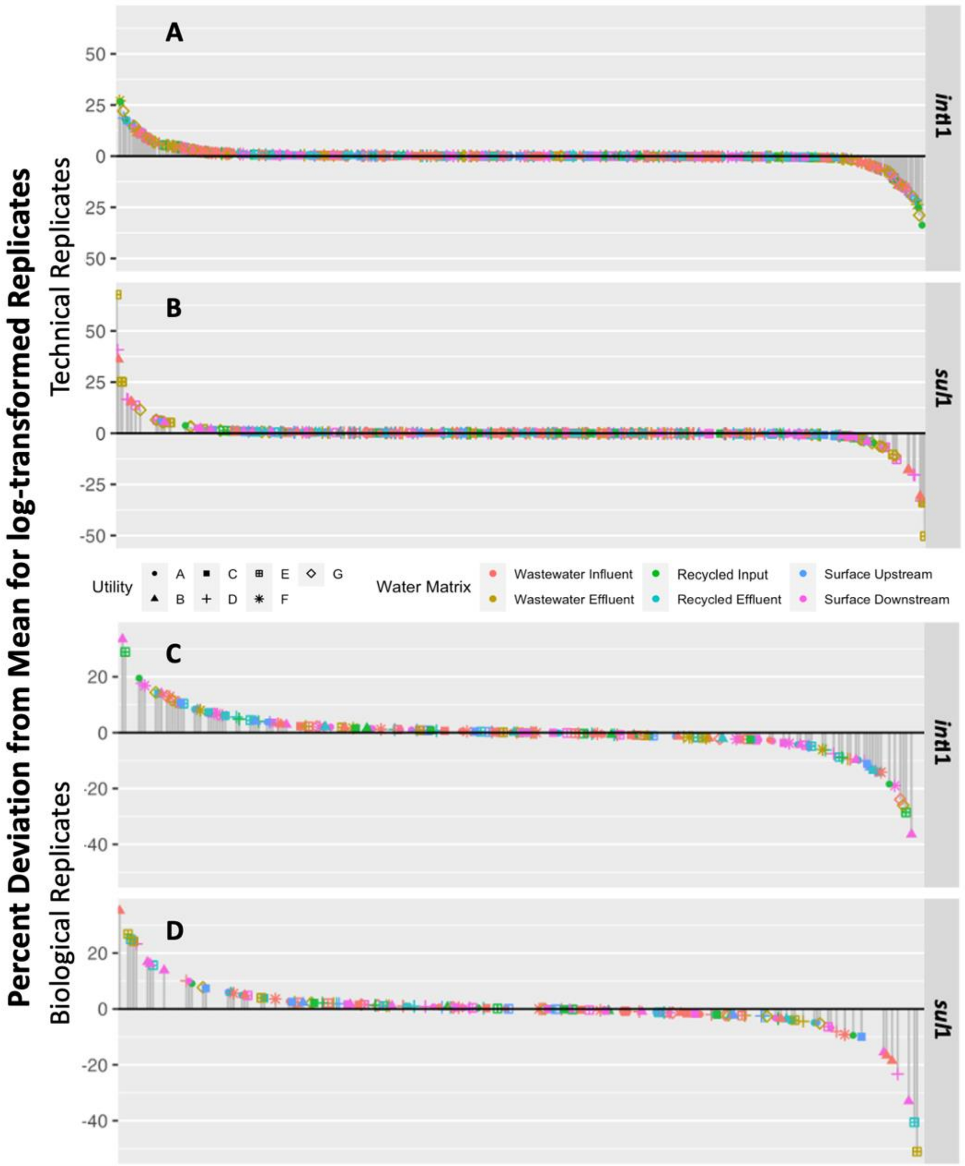

Figure S6: *The percent difference from the mean for each technical replicate for intI1 (A) and sul1 (B) and biological replicate for intI1(C) and sul1 (D) of each sample (samples are unlabeled across the X-axis). Colors indicate water matrix sample; Shapes indicate utility where sample was collected. The mean here is the average of three technical and biological replicates, respectively, collected at the same site and time, and processed at the same laboratory. The horizontal line indicates 0% deviation from the mean as a reference. Replicates analyzed at both Virginia Tech and University of South Florida are all included in this analysis.*

Table S4: Estimates and assumptions for cost analysis of methods for 6 samples (plus biological and technical triplicate for qPCR; plus biological triplicate and technical duplicate for culture)

Method	Target	Lower Estimate, Consumables Only	Upper Estimate, Consumables Only	Estimated Consumable Cost per Sample	Estimated Hours
Culture	total *E. coli* and cefR *E. coli*	$ 120.35	$ 222.63	$20-37	8-9
qPCR	*sul*1 and *int*I1	$ 572.78	$ 645.93	$95-108	9-14
**Assumptions:**	- Culture estimated hours for one technician; qPCR estimated hours range from 1 to 2 technicians at upper estimate.<br>- Lower estimates determined using Virginia Tech's negotiated rates with preferred vendors.<br>- Upper estimates determined using listing prices on the same vendor's sites as viewed without institutional login.				

Table S5: Capital Costs Associated with Methods

Method	Equipment	Use	Lower Estimate	Upper Estimate
**Culture**	Water Bath	Incubation	$591	$3,811
	Incubator	Incubation	$1,677	$8,643
	**Total equipment costs: $2,268 – 12,454**			
**qPCR**	Homogenizer	DNA extraction	$12,607	$16,597
	Mini centrifuge	Preparation of master mix	$178	$450
	Thermal Cycler	Run qPCR	$20,000	$40,000
	Maintenance/year	Thermal cycler	$10,000	$12,000
	Computer	Edit qPCR cycles and download data	$499	$1,500
	**Total equipment costs: $43,284 – 70,547**			
**ddPCR**	Homogenizer	DNA extraction	$12,607	$16,597
	Mini centrifuge	Preparation of master mix	$178	$450

	System with droplet generator, plate reader, and computer	Uniform droplet generation, plate run, download data	$132,610	176,814
	Plate sealer	Uniform seal	$4,293	$4,434
	**Total equipment costs: $149,688 – 198, 295**			

***All three methods utilize a biosafety cabinet and an autoclave, therefore those pieces of equipment were not included in the comparative cost analyses.**

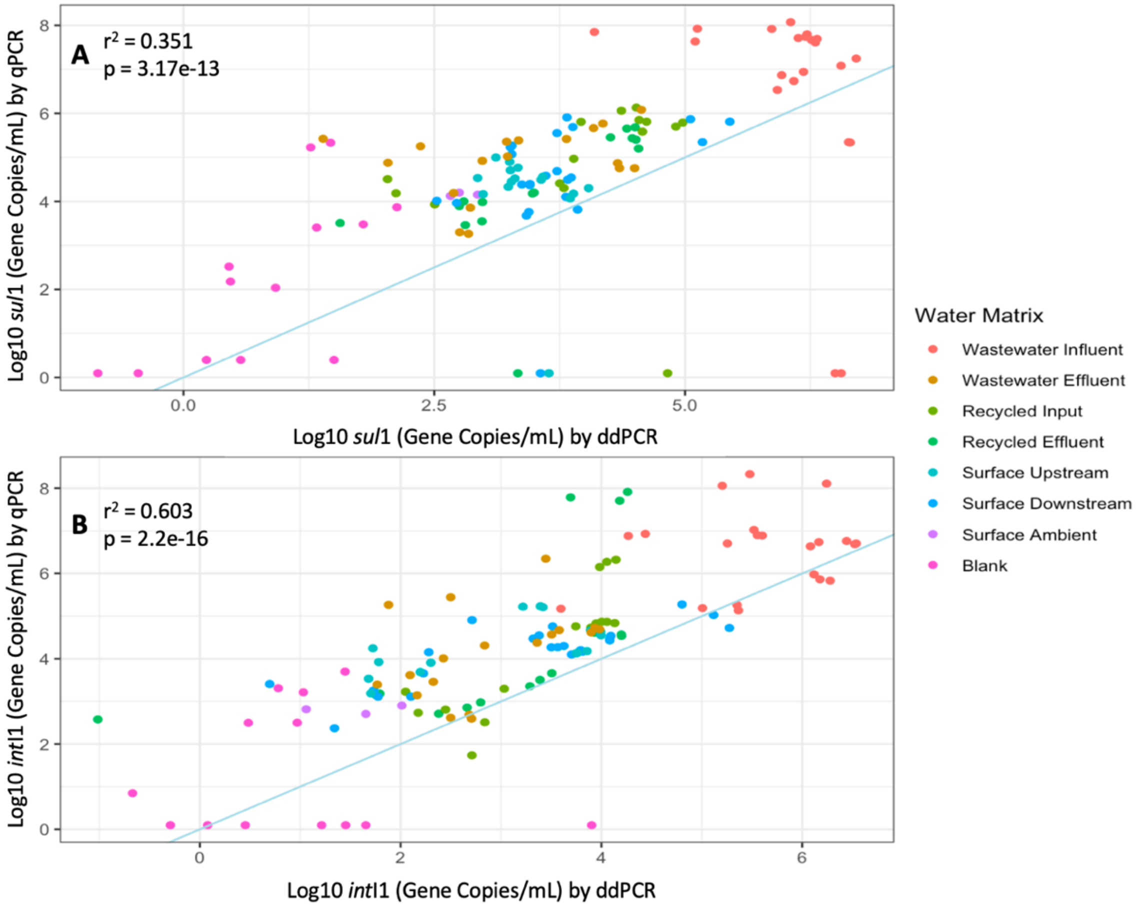

Figure S7: *Correlation of sul*1 (A) and *int*1 *(B) concentrations (gene copies/ml) by qPCR and ddPCR. Color indicates water matrix. Adjusted linear regression r-squared value and p-value are reported. biological replicates analyzed at Virginia Tech are included in this analysis. ddPCR was conducted by partners at US EPA's Office of Research and Development. Light blue lines delineate a 1:1 relationship.*

Correlation:	Wastewater influent	Wastewater Effluent	Recycled Input	Recycled Effluent	Surface Upstream	Surface Downstream
**Total E. coli (VT x USF)**	**p = 0.033 Rho – 0.75**	P = 0.82 Rho = -0.63	-*	P = NA Rho = NA	-*	**P = 0.008 Rho = 1**
**CefR E. coli (VT x USF)**	**P = 0.017 Rho = 0.82**	P = NA Rho = NA	-*	P = NA Rho = NA	-*	**P = 0.043 Rho = 0.92**
**Sul1 (VT x USF)**	P = 0.98 Rho = -0.49	P = 0.56 Rho = - 0.048	-*	**P = 0.026 Rho = 0.61**	-*	P = 0.66 Rho = -0.12
**intI1 (VT x USF)**	P = 0.69 Rho = -0.12	P = 0.06 Rho = 0.57	-*	P = 0.604 Rho = - 0.077	-*	P = 0.532 Rho = -0.077
**Total E. coli x sul1**	P = 0.23 Rho = 0.21	P = 0.71 Rho = -0.20	P = 0.23 Rho = 0.21	P = 0.24 Rho = 0.27	P = 0.53 Rho = 0.0	**P = 0.039 Rho = 0.55**
**CefR E. coli x sul1**	P = 0.47 Rho = 0.024	P = NA Rho = NA	P = 0.47 Rho = 0.024	P = 0.18 Rho = 0.34	P = 0.53 Rho = - 0.051	P = 0.17 Rho = 0.32
**Total E. coli x intI1**	P = 0.631 Rho = - 0.095	P = 0.96 Rho = -0.58	P = 0.63 Rho = - 0.095	**P = 0.032 Rho = 0.64**	P = 0.26 Rho = 0.4	P = 0.59 Rho = -0.078
**CefR E. coli x intI1**	P = 0.33 Rho = 0.13	P = NA Rho = NA	P = 0.33 Rho = 0.13	**P = 0.046 Rho = 0.59**	P = 0.11 Rho = 0.67	**P = 0.036 Rho = 0.56**

Table S6: Spearman correlation for each set of variables for which overall correlations were presented in this paper, with relationship tested within each individual water matrix to assess impact of water matrix variety.

- **recycled influent and surface upstream samples were not collected at USF and therefore no correlation analysis was possible.*
- *Statistically significant correlations bolded for emphasis.*

References

a) Chern, E. C., Brenner, K. P., Wymer, L., & Haugland, R. A. (2009). Comparison of Fecal Indicator Bacteria Densities in Marine Recreational Waters by QPCR. Water Quality, Exposure and Health, 1(3–4), 203–214. https://doi.org/10.1007/s12403-009-0019-2
